# Systemic inflammation-induced adipose tissue remodeling drives psoriasis exacerbation in obesity through epigenetic and immunometabolic dysregulation

**DOI:** 10.7150/thno.116796

**Published:** 2025-07-28

**Authors:** Jinsun Jang, Mijoo Ahn, Jiyeong Jeong, Eun-Hui Lee, Ok-Hee Kim, Seul-A Joo, Seung Eun Baek, Han-Joo Maeng, Yun Hak Kim, In-Sun Hong, Byung-Chul Oh, Ik Soo Kim, Hee Joo Kim, YunJae Jung

**Affiliations:** 1 Department of Health Science and Technology, Gachon Advanced Institute for Health Science & Technology, Gachon University, Incheon, Republic of Korea.; 2 Department of Microbiology, College of Medicine, Lee Gil Ya Cancer and Diabetes Institute, Gachon University, Incheon, Republic of Korea.; 3 Department of Physiology, College of Medicine, Lee Gil Ya Cancer and Diabetes Institute, Gachon University, Incheon, Republic of Korea.; 4 College of Pharmacy, Gachon University, Incheon, Republic of Korea.; 5 Department of Anatomy, School of Medicine, Pusan National University, Yangsan, Republic of Korea.; 6 Department of Biomedical Informatics, School of Medicine, Pusan National University, Yangsan, Republic of Korea.; 7 Department of Molecular Medicine, College of Medicine, Lee Gil Ya Cancer and Diabetes Institute, Gachon University, Incheon, Republic of Korea.; 8 Department of Dermatology, Gachon Gil Medical Center, College of Medicine, Gachon University, Incheon, Republic of Korea

**Keywords:** obesity, psoriasis, adipocytes, macrophages, single-nucleus multiome

## Abstract

**Rationale:** Disruption of adipose tissue homeostasis is increasingly recognized as a key driver of psoriatic inflammation in the context of obesity. However, the mechanisms linking adipose dysfunction to disease severity remain incompletely understood.

**Methods:** We employed an obese mouse model of psoriasis induced by topical imiquimod application or dermal IL-23 injection. Inflammatory profiling from these mice was integrated with multi-omic single-nucleus sequencing targeting RNA and chromatin accessibility to investigate genetic and epigenetic alterations in adipose tissue.

**Results:** Obese mice developed markedly aggravated psoriatic dermatitis following imiquimod treatment, accompanied by increased systemic inflammatory responses and a significant reduction in fat mass. Histological and molecular analyses revealed extensive monocyte-macrophage infiltration into perigonadal adipose tissue, increased expression of pro-inflammatory genes, and upregulation of cell death-associated molecules in obese mice relative to lean counterparts. In contrast, IL-23 injection elicited comparable skin inflammation in both lean and obese mice without inducing adipose tissue loss or systemic inflammation. Multi-omic profiling of imiquimod-treated obese mice revealed genetic and epigenetic changes in adipocytes that promote fatty acid consumption. Furthermore, a shift was observed in macrophage populations—from a lipid-associated subset with active intercellular communication in IL-23-treated mice to disorganized macrophage compartments with monocyte accumulation in imiquimod-treated mice.

**Conclusions:** These findings suggest that obesity sensitizes adipose tissue to homeostatic disruption, establishing it as a critical immunometabolic interface that drives psoriasis exacerbation in response to systemic inflammatory cues.

## Introduction

Psoriasis is a chronic skin disease characterized by epidermal hyperplasia and dermal inflammation 1. Psoriasis involves systemic inflammation beyond the skin and is associated with various complications 2. Metabolic diseases, such as type II diabetes, dyslipidemia, and hypertension, often coexist with psoriasis 3. Additionally, obesity is associated with psoriasis severity and reduced effectiveness of biologics, whereas weight loss enhances the response to both standard systemic therapy and biologics 4. In a murine model of psoriasis, obesity induced by a high-fat diet (HFD) led to increased production of key psoriasis mediators, including IL-17A, IL-22, and S100A8 5. Similar to the weight loss in obese patients with psoriasis, restricting food intake partially can reduce psoriatic inflammation in obese mice 6.

Obesity and associated metabolic disorders are serious health problems 7. However, obesity does not necessarily equate to a negative metabolic condition, particularly when excess energy is stored in metabolically healthy adipose tissue 8. Adipose tissue is pivotal in maintaining metabolic homeostasis by storing excess lipids and secreting regulatory adipokines 9. In the absence of adipose tissue inflammation, even individuals with obesity may remain metabolically healthy 10. However, an inflammatory response within adipose tissue, marked by immune cell infiltration, particularly by macrophages, disrupts this homeostatic balance 11, 12. This inflammatory remodeling leads to the release of fatty acids and pro-inflammatory cytokines, contributing to systemic metabolic dysregulation 13. Emerging evidence suggests that such disruption of adipose tissue homeostasis contributes to the severity of chronic inflammatory diseases such as psoriasis 11, 14. Nonetheless, the mechanisms by which adipose dysfunction exacerbates psoriasis in the context of obesity remain incompletely understood.

Advances in single-cell RNA-sequencing (RNA-seq) have identified diverse cell types within adipose tissue and revealed their complex interactions 15. Furthermore, the role of obesity-specific subsets of resident or infiltrated immune cells and mesenchymal stem cells has been emphasized in the regulation of metabolic disorders 16, 17. Despite the recognition of obesity-associated epigenetic modifications, our understanding of the epigenetic landscape of individual cell types within inflamed, obesity-adapted adipose tissue remains limited. Integrating gene expression and epigenetic profiling at the single-cell level provides a powerful approach to uncover the mechanisms underlying obesity-associated exacerbation of inflammatory diseases.

In the present study, we investigated the impact of HFD-induced obesity in a murine model of psoriasis induced by topical imiquimod application or dermal IL-23 injections. We assessed skin inflammation, circulating inflammatory mediators, and adipose tissue alterations. Additionally, we integrated inflammatory profiling from these mice and compared transcriptomic and epigenetic alterations in adipose tissue at the single-nucleus level to gain insights into the mechanisms by which adipose tissue dysfunction contributes to the exacerbation of obesity-associated psoriasis.

## Methods

### Mice

C57BL/6 male mice purchased from Orient Bio (Gyeonggi-do, Korea) were maintained at standard temperature and humidity in a specific pathogen-free environment. All animal procedures were reviewed and approved by the Institutional Animal Care and Use Committee (IACUC) of the Lee Gil Ya Cancer and Diabetes Institute at Gachon University (Number: LCDI-2020-0113). All procedures were conducted in compliance with the ARRIVE guidelines and the *Guide for the Care and Use of Laboratory Animals* (National Research Council, 2011). Mice were fed a 60% HFD (Research Diets, New Brunswick, NJ, USA) or a 5.0% fat chow diet (Chow, LabDiet, St. Louis, MO, USA) for 12 weeks. Body weight was measured weekly. At sacrifice, all mice were weighed, and blood samples, skin, spleen, perigonadal adipose tissue, and liver tissue were collected for subsequent analyses.

### Animal model of experimental psoriasis

A topical dose of 62.5 mg of imiquimod cream (5%, Aldara™; 3M Pharmaceuticals, Maplewood, MN, USA) or vehicle cream (Vaseline; Unilever, Rotterdam, Netherlands) was applied to the shaved back daily for 4 days. To induce psoriatic skin inflammation by IL-23, mice were injected with 1 μg recombinant mouse IL-23 (R&D Systems, Minneapolis, MN, USA) in phosphate-buffered saline (PBS) daily at a labeled location on the shaved back for 5 days. Mice were assessed daily as previously described 18.

### Histology

Skin specimens and perigonadal adipose tissue were fixed in 10% neutral buffered formalin (BBC Biochemical, Mount Vernon, WA, USA) and embedded in paraffin. Multiple 4 μm-thick sections were stained with hematoxylin and eosin. For double immunofluorescence staining, sections from perigonadal adipose tissue were incubated overnight with TREM2 mAb or CCR2 mAb and F4/80 mAb (all from Abcam, Cambridge, UK). Sections were washed with phosphate-buffered saline and then incubated with Alexa Fluor 488-linked goat anti-rat IgG (H&L) secondary antibody and Alexa Fluor 647-linked goat anti-rabbit IgG (H&L) secondary antibody (all from Abcam) for 1 h. After 10 min of staining with 4',6-diamidino-2-phenylindole (Thermo Fisher Scientific, Waltham, MA, USA), slides were mounted using ProLong Gold Antifade (Molecular Probes, Eugene, OR, USA) and visualized using a DM6 B microscope equipped with a DFC7000T camera (Leica, Wetzlar, Germany). The thicknesses of subcutaneous fat and fluorescence signals were measured using i-SOLUTION™ (IMT i-Solution Inc., Vancouver, BC, Canada). The size of perigonadal adipocytes was estimated using Adiposoft software (National Institutes of Health, Bethesda, MD, USA).

### Quantitative PCR

RNA was isolated and purified using an RNeasy mini kit (Qiagen, Hilden, Germany). The purified RNA was processed with DNase I (New England Biolabs, Ipswich, MA, USA) to remove genomic DNA. Complementary DNA (cDNA) was synthesized using an iScript™ cDNA synthesis kit (Bio-Rad Laboratories, Hercules, CA, USA). Quantitative PCR was performed using iQ SYBR^®^ Green Supermix on a CFX Connect™ real-time PCR detection system (all from Bio-Rad Laboratories). Relative gene expression was determined using the 2^-ΔΔCt^ method, and the glyceraldehyde 3-phosphate dehydrogenase gene (*Gapdh*) served as an invariant control. The primer sequences are presented in **Table S1**.

### Protein array analysis

The serum profile of 40 different cytokines was assessed using a Mouse Cytokine Array Kit (R&D Systems) following the manufacturer's instructions. Chemiluminescence signals corresponding to the amount of cytokines were detected using an ImageQuant LAS 4000 biomolecular imager (GE Healthcare Bio-Sciences AB, Uppsala, Sweden). Signal quantification was performed using the HLimage++ toolkit (Western Vision Software, Salt Lake City, UT, USA).

### RNA extraction, library construction, and sequencing

Total RNA concentration was calculated using Quant-IT RiboGreen (Invitrogen, Waltham, MA, USA). To assess the integrity of the total RNA, samples were run on the TapeStation RNA screentape (Agilent, Santa Clara, CA, USA) with an RNA integrity threshold value of ≥7. A library was independently prepared with 1 µg total RNA for each sample using the TruSeq Stranded mRNA Sample Prep Kit (Illumina, San Diego, CA, USA). The libraries were amplified through PCR and then quantified using KAPA Library Quantification Kit (Kapa Biosystems, Wilmington, MA, USA) and qualified using the TapeStation D1000 ScreenTape (Agilent). Indexed libraries were then submitted to NovaSeq sequencing (Illumina). Paired-end (2 × 100 bp) sequencing was performed by Macrogen (Seoul, Korea).

### Sequence annotation and statistical analysis of gene expression

The raw reads were preprocessed to remove low-quality and adapter sequences. The processed reads were aligned to the mm10 mouse genome reference using HISAT v2.1.0 19. Thereafter, aligned reads were assembled into transcripts using StringTie v2.1.3b, and their abundance was estimated 20, 21. Genes with a read count of zero in at least one sample were excluded. To facilitate log_2_ transformation, 1 was added to each read count value of filtered genes. Filtered data were log_2_-transformed and subjected to relative log expression normalization. The statistical significance of the differential expression data was determined using nbinomWaldTest with DESeq2 and fold change. The null hypothesis that no difference exists among groups was considered.

### Gene set enrichment analysis (GSEA)

GSEA of RNA-seq data was performed using the GSEA v4.3.2 software provided by the Broad Institute (Cambridge, MA, USA) as previously described 22. Enrichment analysis was performed using the hallmark gene sets of the MsigDB database. To determine the enrichment of ontology gene sets (C5.all.v2022.1), mouse gene symbols were remapped to human orthologs. Leading-edge analysis was performed to determine the overlapping gene sets. Selected gene sets with *p* < 0.05 and a false discovery rate < 0.25 were considered.

### Western blotting

Cell lysates were prepared in ice-cold tissue lysis RIPA buffer containing a protease inhibitor cocktail and phosphatase inhibitor cocktail (all from Thermo Fisher Scientific). The protein content was measured using a BCA Protein Assay Kit (Thermo Fisher Scientific). Proteins from tissue lysates were resolved using SDS-PAGE and transferred to a polyvinylidene difluoride membrane. After 1 h of blocking using 5% bovine serum albumin (BSA) in Tris-buffered saline containing 0.1% Tween-20 (TBST), the membrane was incubated overnight at 4 °C with antibodies against cleaved-caspase-3 (c-caspase-3), caspase-3, phosphorylated receptor-interacting protein kinase 3 (pRIP3), and β-actin (all from Cell Signaling Technology, Danvers, MA, USA), RIP3 (ProSci, Poway, CA, USA), and heme oxygenase-1 (HO-1; Abcam) at 1:1000 dilution in TBST. After three TBST washes, membranes were incubated with a secondary antibody for 1 h at room temperature. Chemiluminescence was performed using ECL Western Blotting Substrate (Thermo Fisher Scientific).

### Single-nucleus preparation

Nuclei isolation from frozen mouse adipose tissue was performed following a modified protocol for single-cell multiome assay for transposase-accessible chromatin (ATAC) analysis. Frozen mouse adipose tissues were minced on dry ice and transferred to a gentle MACS C tube (Miltenyi Biotec, Bergisch Gladbach, Germany) with chilled TST buffer 23 containing 0.03% Tween 20, 0.01% BSA, 146 mM NaCl, 1 mM CaCl_2_, 21 mM MgCl_2_, and 10 mM Tris-HCl pH 7.5, prepared in ultrapure water. Tissues were dissociated using the gentleMACS Dissociator (Miltenyi Biotec), incubated on ice for 10 min, and filtered through a 40-µm cell strainer into a single-nucleus suspension. Nuclei were centrifuged at 500 ×g for 5 min at 4 °C, resuspended in chilled nuclei resuspension buffer containing 0.04% BSA and 0.2 U/μL RNase inhibitor in PBS. Nuclei were further purified and enriched using antinuclear microbeads (Miltenyi Biotec). The viability and concentration of nuclei were determined using Countess 3 (Thermo Fisher Scientific) with trypan blue staining.

### Hashing of nuclei by hash-tag antibody staining

Isolated nuclei were resuspended in a staining buffer containing 2% BSA and 0.01% Tween in PBS and incubated with Fc receptor-blocking solution (FcX; BioLegend, San Diego, CA, USA) for 5 min on ice. The nuclei of each sample were incubated with a specific hash-tag antibody (TotalSeq™-A0451~8, BioLegend, **Table S2**) for 20 min on ice and then washed thrice with staining buffer. Following centrifugation at 500 ×g for 5 min at 4 °C, hash-tagged nuclei were resuspended in the appropriate buffer for downstream analysis and combined to multiplex three or four samples, each containing an equal number of nuclei.

### Single-nucleus RNA- and ATAC-seq

Single-nucleus RNA- and ATAC-seq were performed using a Chromium Next GEM Single Cell Multiome ATAC and Gene Expression Kit (10x Genomics, Pleasanton, CA, USA). Nuclei were incubated at 37 °C for 60 min in a VeritiPro 96-Well Thermal Cycler (Thermo Fisher Scientific) with Transposition Mix for ATAC-seq, followed by GEM generation on the Chromium instrument (10x Genomics). Following reverse transcription and cleanup, barcoded ATAC fragments, cDNA, and hash-tag barcoding antibody-oligo (HTO) were amplified with the pre-amplification mix with 1 µL of 0.2 µM HTO-targeting additive primer (**Table S2**). Separate ATAC, cDNA, and HTO libraries were constructed, cleaned, and processed using the MGIEasy Universal Library Conversion Kit (App-A) (MGI, Shenzhen, China). Sequencing was performed on the DNBSEQ-G400 (MGI).

### Single-nucleus ATAC, RNA, and HTO sequence extraction and alignment

Sequencing data were generated with a DNBSEQ-G400 system. Fastq files containing cDNA and HTO data were separated using splitBarcode v.2.0.0 (MGI) by sample index sequences. The reads of Fastq files were trimmed with Trimmomatic v.0.39. Single-nucleus ATAC- and RNA-seq data were aligned and counted using Cell Ranger ARC count v.2.0.2 (10x Genomics). HTO data were preprocessed using CITE-seq-Count v.1.4.5.

### Single-nucleus RNA-seq data processing

Single-nucleus RNA-seq data were processed using Seurat v.5.0.1 for demultiplexing, quality control, normalization, integration, dimensionality reduction, and clustering. After filtering low-quality nuclei, datasets were normalized and scaled using the “SCTransform,” and data integration was performed using the reciprocal PCA method in Seurat, in which shared highly variable features were used to identify integration anchors across datasets. Nuclei were clustered using the Louvain algorithm and visualized using Uniform Manifold Approximation and Projection (UMAP). Cell types were identified using SingleR v.1.8.1, and differentially expressed genes (DEGs) were analyzed with the “FindMarkers” function. Gene set enrichment analysis was performed using clusterProfiler v.4.2.2. Cell-cell communication was estimated through the CellChat algorithm (v.1.6.1). For trajectory analysis, Velocyto v.0.17.17 and scVelo v.0.3.1 were used to estimate velocities and visualize them in UMAP coordinates, with latent time estimated using scVelo functions.

### Single-nucleus ATAC-seq data processing

Data processing was conducted using Seurat v.5.0.1 and Signac v.1.12.0, for merging, peak calling, and integrating the RNA and ATAC metadata. A common peak set was created with the GenomicRanges library, and the peaks were quantified using MACS2. Multiple modalities of mRNA levels and ATAC peaks were integrated using the weighted-nearest neighbor method. Alternatively, RNA-derived metadata was merged with ATAC-derived data, and each modality was projected using RNA-derived UMAP and cluster information in single-nucleus ATAC-seq analysis. Differentially accessible regions (DARs) were extracted using a Wilcoxon rank sum test and a latent variable in the Signac package. Putative transcription factor binding motifs enriched in DARs were identified using JASPAR 2020. Co-accessible regions were calculated using Cicero v.1.3.9 and annotated with ChIPseeker v.1.30.3. The annotated peaks and co-accessibility score were analyzed for gene ontology over-representation using clusterProfiler v.4.2.2.

### Chromatin immunoprecipitation (ChIP) and quantitative PCR

ChIP assays were conducted using the High-Sensitivity ChIP Kit (Abcam), following the manufacturer's instructions. In brief, adipose tissue samples were cross-linked with 2 mM DSG (30 min) and 1% formaldehyde (15 min). After glycine quenching, nuclei were isolated in lysis buffer containing protease inhibitors, and chromatin was sheared through sonication to 200-500 bp fragments. After centrifugation and concentration, chromatin was incubated overnight with antibodies against POU4F2, POU3F1, NR3C1, KLF5, SP1, EGR1, or IgG (as a control), as listed in **Table S3**. DNA was eluted, treated with RNase A and proteinase K, and purified. Enriched DNA was incubated overnight at 60 °C for 30 min to reverse crosslinking, then quantified and analyzed through qPCR with gene-specific primers (listed in **Table S4**).

### Graphical illustrations

Schematics of experimental workflows were created using a licensed version of Biorender.com.

### Statistical analyses

Experiments were performed in duplicate or triplicate, with two independent tests for each assay, except sequencing analysis. Two-group comparisons used a two-tailed unpaired Student's *t*-test or Mann-Whitney test. Statistical differences between groups were examined using one-way ANOVA with Tukey's post hoc or Kruskal-Wallis test with Dunn's multiple comparisons. For multiple comparisons, two-way ANOVA with Bonferroni's test was used. A *p*-value < 0.05 was considered significant. GraphPad Prism 10.3.1 (GraphPad, San Diego, CA, USA) was used for the data analysis.

## Results

### Obesity aggravates psoriatic dermatitis with a systemic increase of inflammatory mediators

**Figure 1:**
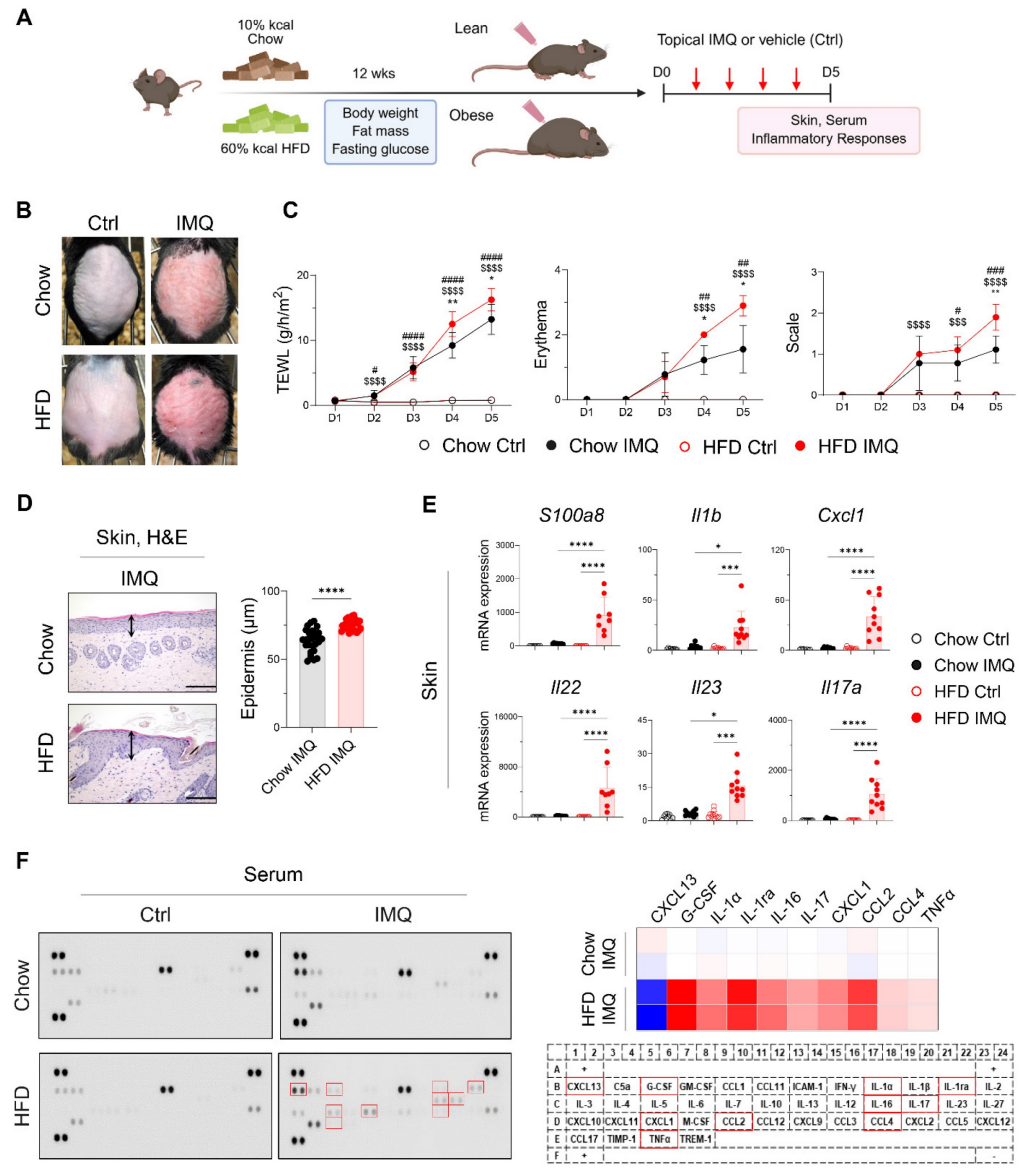
** Obesity exacerbates imiquimod (IMQ)-induced psoriatic dermatitis.** (A) Schematic diagram of experimental psoriasis induction through topic IMQ application. (B) Photographs of skin. (C) Transepithelial water loss (TEWL), erythema, and scale of IMQ and vehicle (Ctrl) treated skin of mice fed the high-fat diet (HFD) or chow diet (Chow) (n = 9-10 per group). (D) Hematoxylin and eosin-stained images (left) and epidermal thickness (right). Three distinct regions of each section were analyzed. Scale bars = 124.5 μm. (E) Quantitative PCR analysis of inflammatory mediators in the skin. (F) Representative image of the cytokine array membrane (left), heat map displaying quantified density (top right), and schematic representation of the coordinates of the mouse cytokine array (bottom right). Data are presented as the mean ± standard deviation (SD). *, Chow IMQ vs. HFD IMQ; #, Chow Ctrl vs. Chow IMQ; $, HFD Ctrl vs. HFD IMQ. *p < 0.05, **p < 0.01, ***p < 0.001, and ****p < 0.0001 using two-way ANOVA (C), unpaired *t*-test (D), or one-way ANOVA (E).

After confirming that the HFD-fed mice gained more body weight and fat mass and displayed insulin resistance compared with the chow diet (Chow)-fed mice (**Figure S1A**), we topically applied imiquimod to the backs of the Chow- and HFD-fed mice (**Figure 1A**). Compared with the Chow-fed group, clinical symptoms were more severe, and the epidermis was significantly thicker in HFD-fed mice (**Figure 1B**-**D**). Consistent with these findings, imiquimod-treated obese mice showed significantly increased expression of *S100a8*, *Il1b*, *Cxcl1*, *Il22*, *Il23*, and *Il17a* in lesional skin compared to lean mice (**Figure 1E**). Furthermore, imiquimod-treated obese mice had high serum levels of innate cell growth factors, including granulocyte-colony stimulating factor and inflammatory cytokines (IL-1α, IL-16, IL-17, and tumor necrosis factor-alpha [TNFα]), along with increased chemokine levels, especially C-X-C motif ligand 1 (CXCL1), C-C motif ligand 2 (CCL2), and CCL4, and decreased levels of CXCL13 compared with the serum levels in Chow-fed mice (**Figure 1F**). Importantly, these serum levels of immune mediators remained largely unchanged in obese mice without psoriatic inflammation (**Figure S2**). Consistent with these findings, we observed an increase in the serum levels of imiquimod, and this increase was more prominent in HFD-fed mice with psoriatic inflammation (**Figure S1B**). These observations suggest that obesity alone does not exacerbate psoriasis, but that the specific clinical features of psoriasis drive its aggravation in obese mice.

### Psoriatic dermatitis associated with systemic inflammation reduces adipose tissue mass

**Figure 2:**
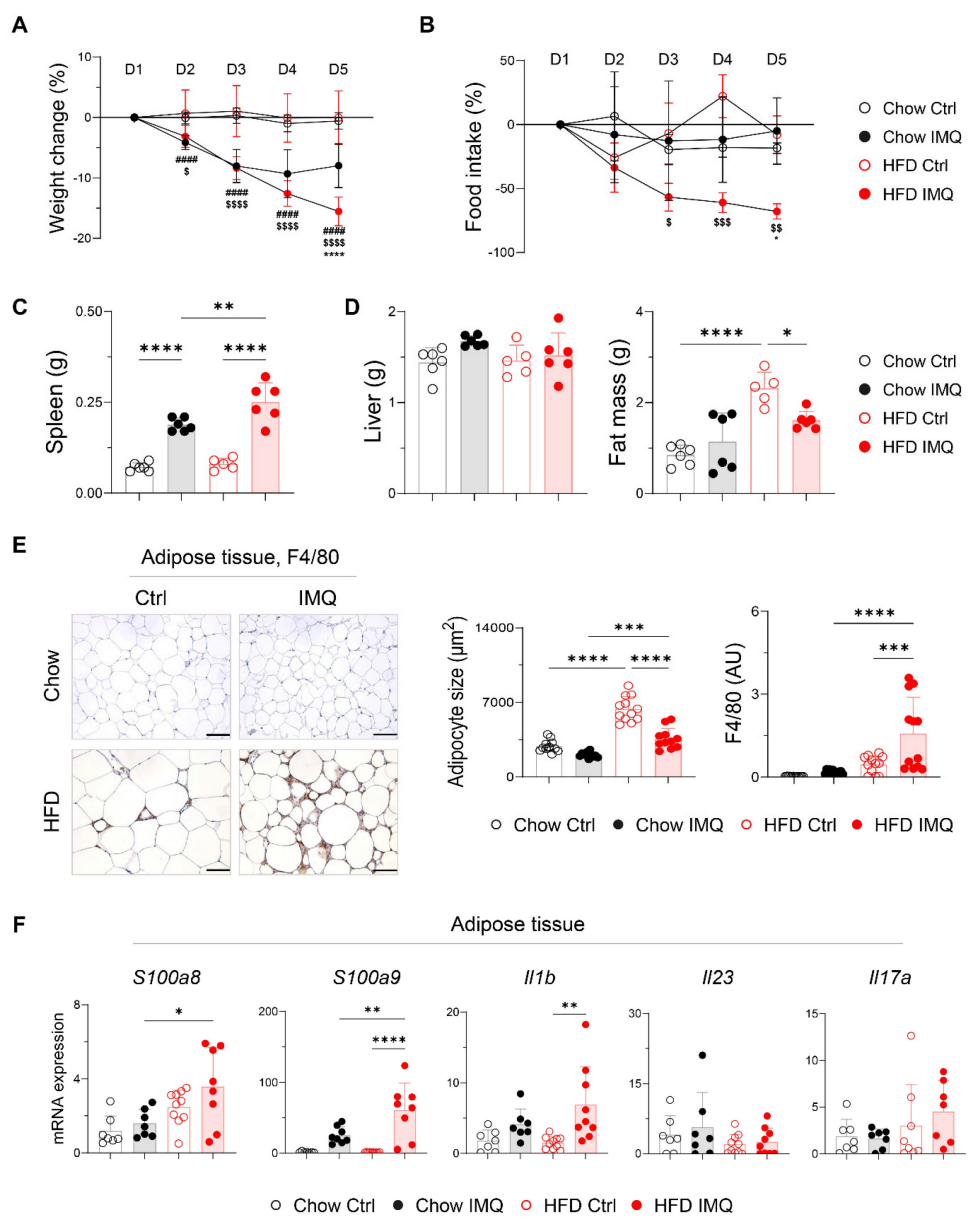
** Decreases in body weight and fat mass are pronounced in obese mice with imiquimod (IMQ)-induced psoriatic dermatitis.** (A) Body weight change (n = 12-13 per group). (B) Food intake (n = 4-5 per group). (C) Weight of the spleen. (D) Weight of the liver (left) and perigonadal adipose tissue mass (right). (E) Immunohistochemical staining images of F4/80 (left), average size of adipocyte (middle), and intensity quantification of F4/80 (right) in perigonadal adipose tissue. Scale bars = 124.5 μm. Three distinct regions of each section were analyzed for histology quantification. (F) Quantitative PCR analysis of inflammatory mediators in perigonadal adipose tissue. Data are presented as the mean ± SD. *, Chow IMQ vs. HFD IMQ; #, Chow Ctrl vs. Chow IMQ; $, HFD Ctrl vs. HFD IMQ. *p < 0.05, **p < 0.01, ***p < 0.001, and ****p < 0.0001 using two-way ANOVA (A and B) or one-way ANOVA (C-F).

Given that dermal imiquimod induces systemic inflammation 24, 25, we hypothesized that obesity combined with systemic inflammation exacerbates psoriatic dermatitis. Imiquimod administration caused immediate body weight loss, which was more pronounced in obese mice, indicating an intensified systemic inflammatory response (**Figure 2A**). This was further supported by a reduction in food intake and splenic enlargement (**Figure 2B**-**C**). Although liver weights remained similar, obese mice showed a significant decrease in perigonadal adipose tissue mass, unlike lean mice (**Figure 2D**). Consistently, imiquimod treatment led to over 40% reduction in adipocyte size in obese mice (**Figure 2E**), along with decreased subcutaneous fat thickness (**Figure S3A**). Remarkably, a histologic crown-like structure (CLS), characterized by the clustering of F4/80^+^ monocytes and macrophages surrounding a dying adipocyte 26, was frequently observed in the adipose tissue of obese mice with psoriatic inflammation (**Figure 2E**). In parallel, the expression of genes encoding innate inflammatory mediators was significantly upregulated in the adipose tissue from these mice (**Figure 2F**). Notably, *Il23* and *Il17a* expression remained unchanged either by diet or imiquimod treatment, suggesting that adipose tissue inflammation in obese mice with psoriasis proceeds independently of direct activation of the IL-23/IL-17 axis.

### Imiquimod-induced psoriatic dermatitis in obese mice elicits adipose tissue inflammation

**Figure 3:**
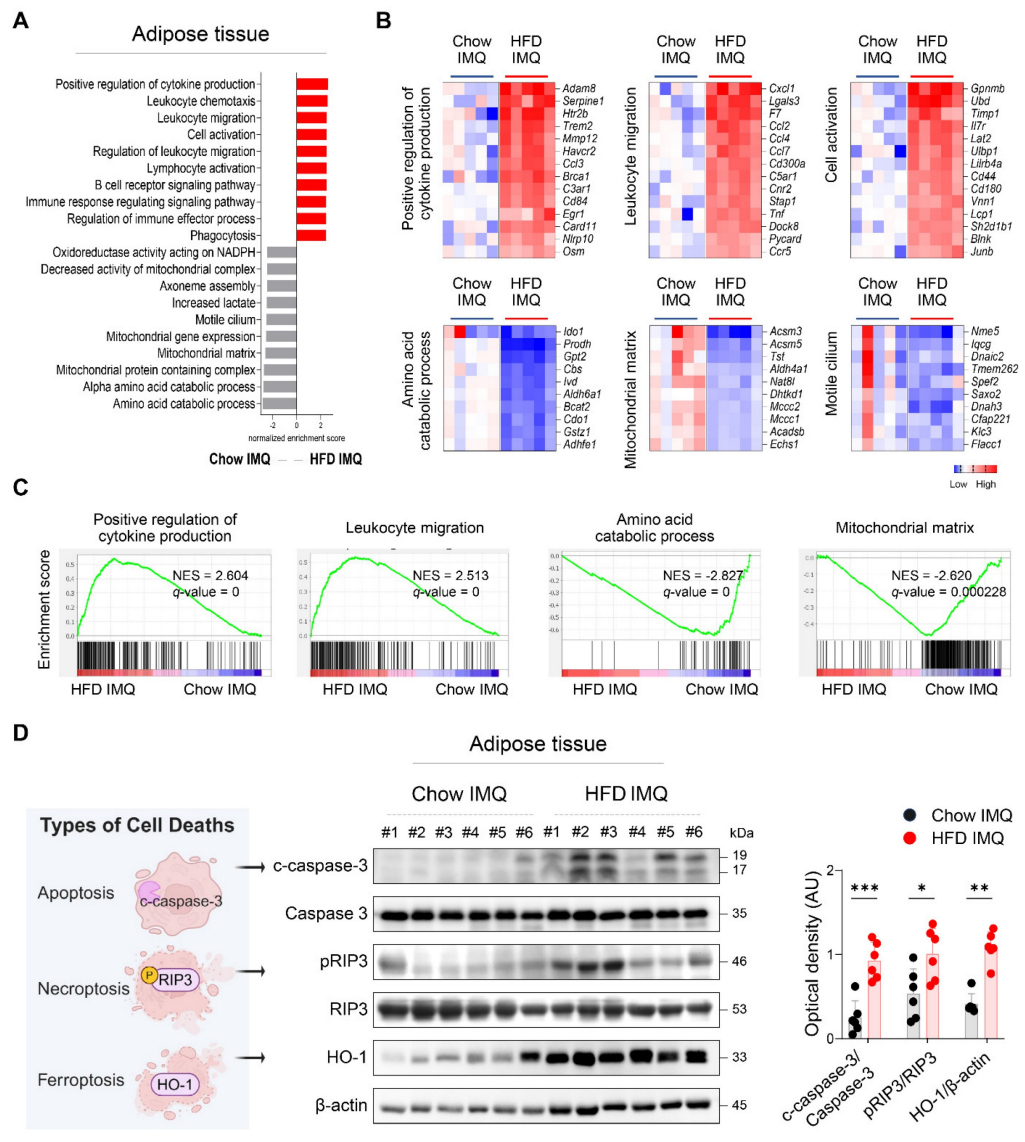
** Expression of inflammatory mediators and death-associated molecules is increased in adipose tissue of obese mice with imiquimod (IMQ)-induced psoriatic dermatitis.** (A) Gene ontology analysis of enriched genes identified in perigonadal adipose tissue using gene set enrichment analysis in mice treated with IMQ. (B and C) Selected gene sets (B) and enrichment plots (C) that were differentially expressed in the perigonadal adipose tissue of mice treated with IMQ. Heat map of the log_2_ fold change to the geometric mean of fragments per kilobase million + 0.01. Normalized enrichment score (NES) and false-discovery rate (*q*) are used to assess significance, with a threshold of *q* <0.05. (D) Schematic diagram of cell death (left), representative western blot (middle), and quantification (right) of c-caspase-3, pRIP3, and HO-1 proteins. β-actin was used as an internal control. Data are presented as the mean ± SD. *p < 0.05, **p < 0.01, and ***p < 0.001 using the unpaired *t*-test (c-caspase-3/caspase-3 and p-RIPK3/RIPK3 in D), or Mann-Whitney test (HO-1/β-actin in D).

To gain deeper insight into the changes in adipose tissue, we compared the transcriptome of adipose tissue from imiquimod-treated obese mice and lean mice. GSEA revealed that adipose tissue from lean mice was enriched for gene sets associated with metabolic processes, whereas that from obese mice displayed enrichment in pathways related to inflammatory responses, including cytokine production, leukocyte migration, and cell activation (**Figure 3A**-**C**). Differential gene expression analysis further identified several innate immune-related genes, such as *Osm*, *Tnf*, and *Pycard* 27-29, as among the most highly upregulated in the adipose tissue of obese mice with psoriasis (**Figure 3B**), suggesting robust activation of innate immune networks in this context.

Given that regulated cell death contributes tissue damage and inflammation 30, the decreased adipocyte size observed in obese mice with psoriatic inflammation, together with an upregulated inflammatory response, suggests that adipocytes in this context may experience accelerated cell death. Consistently, significantly increased levels of c-caspase-3, pRIP3, and HO-1, markers associated with apoptosis, necroptosis, and ferroptosis, respectively 31, were detected in obese mice compared to Chow-fed controls with psoriatic inflammation (**Figure 3D**).

### IL-23-induced psoriatic dermatitis is not exacerbated in obese mice

**Figure 4:**
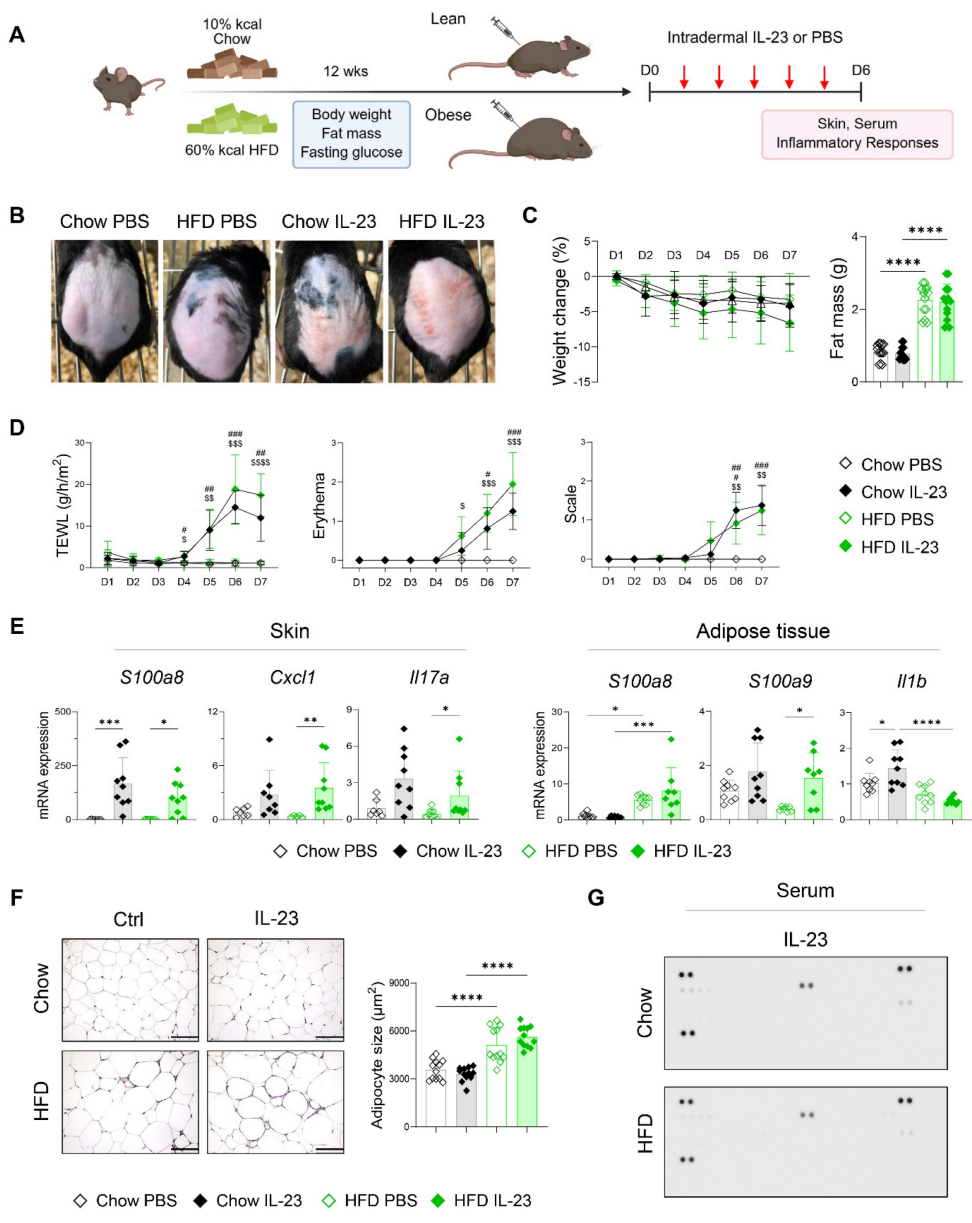
** Intradermal IL-23 injection does not exacerbate psoriatic dermatitis or induce adipose tissue damage in obese mice. (**A) Schematic diagram of experimental psoriasis induction through intradermal IL-23 injection. (B) Photographs of skin. (C) Body weight change and weight of perigonadal adipose tissue mass (n = 8-10 per group). (D) Transepithelial water loss (TEWL), erythema, and scale (n = 9-10 per group). (E) Quantitative PCR analysis of inflammatory mediators in the skin (left) and perigonadal adipose tissue (right). (F) Hematoxylin and eosin-stained images (left) and average size (right) of perigonadal adipose tissue. Scale bars = 124.5 μm. Three distinct regions of each section were analyzed for histological quantification. (G) Representative image of the cytokine array membrane. Data are presented as the mean ± SD. *, Chow IL-23 vs. HFD IL-23; #, Chow PBS vs. Chow IL-23; $, HFD PBS vs. HFD IL-23. *p < 0.05, **p < 0.01, ***p < 0.001, and ****p < 0.0001 using Kruskal-Wallis test (C and skin *Cxcl1* and *Il17a* in E), two-way ANOVA (D), or one-way ANOVA (E and F).

We hypothesized that the inflammatory damage to adipose tissue in obesity-associated psoriasis arises from systemic features of psoriasis. Following the observation of an imiquimod-induced increase in cutaneous blood perfusion, which was not evident in mice receiving an intradermal IL-23 injection (**Figure S4**), we compared disease phenotypes in lean and obese mice after intradermal IL-23 injection (**Figure 4A**). IL-23 injection induced comparable psoriatic skin inflammation in both lean and obese mice, without accompanying weight loss or reduction in fat mass (**Figure 4B-D**). The expression of inflammatory mediators was similarly upregulated in the skin lesions of both groups following IL-23 injection (**Figure 4E**). In contrast to imiquimod application, IL-23 injection resulted in decreased expression of *S100a9* and *Il1b* in the adipose tissue of obese mice compared with lean controls (**Figure 4E**). Consistent with these findings, adipocyte size (**Figure 4F**), subcutaneous fat thickness (**Figure S3B**), and serum levels of immune mediators (**Figure 4G**) and IL-23 (**Figure S3C**) remained unchanged in obese mice injected with IL-23. Moreover, a decrease in high-density lipoprotein and free fatty acids, indicating inflammation-associated lipid utilization 32, was observed only in imiquimod-treated obese mice, but not in those injected with IL-23 (**Figure S5**). Collectively, these findings suggest that intradermal IL-23 injection, which does not elicit systemic inflammation or adipose tissue damage, fails to exacerbate psoriatic inflammation in obese mice.

### Single-nucleus sequencing reveals upregulation of catabolic pathways in adipocytes of obese mice with imiquimod-induced psoriatic dermatitis

**Figure 5:**
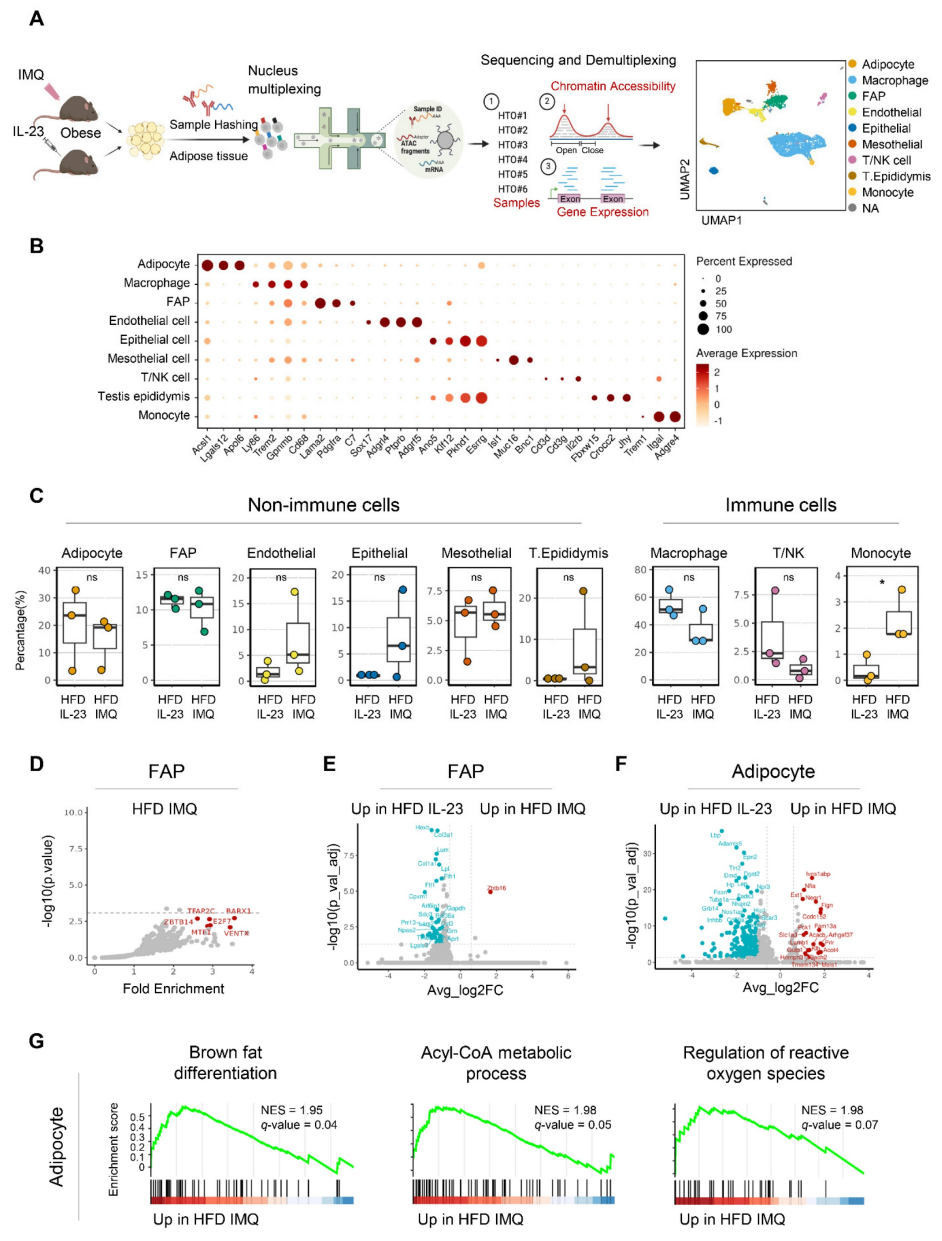
** Expression of genes implicated in catabolic pathways is upregulated in adipocytes of obese mice with imiquimod (IMQ)-induced psoriatic dermatitis.** (A) Schematic of single-nucleus RNA- and assay for transposase-accessible chromatin (ATAC)-seq of perigonadal adipose tissue. The Uniform Manifold Approximation and Projection (UMAP) plot displays deconvoluted adipose tissue cell types. (B) Dotplot of the cell composition of adipose tissue based on the differentially expressed genes across clusters. (C) Proportional differences of annotated cell types upon treatments. (D) DNA sequence motif analysis illustrating differential chromatin accessibility. The dotted line represents the significance threshold. (E) Volcano plot of differentially expressed genes in fibroblast and adipocyte progenitor cells (FAP). (F) Volcano plot of differentially expressed genes in adipocytes. (G) Running score plot of the enrichment of upregulated genes in adipocytes of IMQ-treated group within specific gene clusters. Normalized enrichment score (NES) and false-discovery rate (*q*) are used to assess significance, with a threshold of *q* <0.05. Data are presented as the mean ± SD. ns: *p* > 0.5 and *p < 0.05 using the unpaired *t*-test.

The distinct adipose tissue responses observed in obese mice with imiquimod- versus IL-23-induced psoriatic dermatitis prompted a detailed investigation into the underlying molecular mechanisms. Accordingly, we performed single-nucleus RNA and ATAC sequencing on adipose tissue from obese mice treated with either imiquimod- or IL-23 treatment (**Figure 5A**). Nine cell types were identified from 6,574 nuclei in perigonadal adipose tissue (**Figure 5A**-**B**). The samples were demultiplexed by HTO identity, and the cell type proportions were compared (**Figure 5C**). Although most types showed no significant changes, a notable increase in the monocyte subset was observed in imiquimod-treated obese mice (**Figure 5C**). Following the observed decrease in fat mass in imiquimod-treated obese mice, we compared the genomic and epigenomic profiles of adipocytes and fibroblast and adipocyte progenitors (FAP). No significant changes in DEGs or DARs were found in FAP across the treatments, except for a reduction in obesity-related genes in imiquimod-treated FAP (**Figure 5D**-**E**). Consequently, specific gene signatures were not enriched in FAP, implying that progenitor cell alterations might not account for the reduced fat mass observed following imiquimod treatment. In IL-23-treated adipocytes, the upregulated genes included typical obesity markers such as *Lep*, which encodes leptin (**Figure 5F**). In contrast, gene sets involved in fatty acid expenditure, such as brown fat differentiation and acyl-CoA metabolism, were enriched in adipocytes from imiquimod-treated obese mice (**Figure 5G**). Despite the marginal significance, gene sets regulating reactive oxygen species were enriched in the imiquimod-treated mice, suggesting adipocyte damage (**Figure 5G**).

### Chromatin reorganization in adipocytes of obese mice with imiquimod-induced psoriatic dermatitis facilitates catabolic metabolism

**Figure 6:**
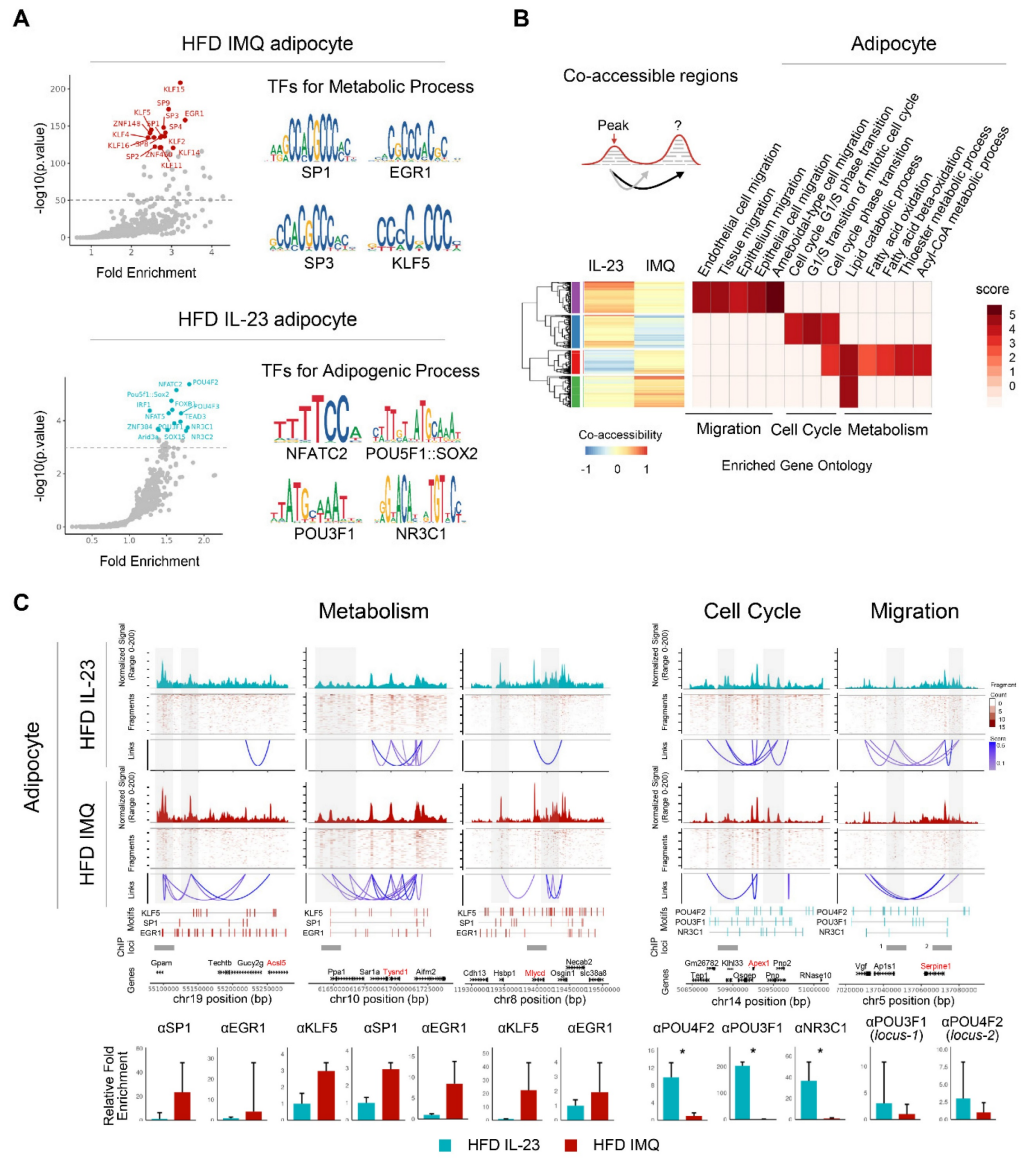
** Chromatin accessibility is reorganized to promote catabolic metabolism in adipocytes of obese mice with imiquimod (IMQ)-induced psoriatic dermatitis.** (A) DNA sequence motif analysis of differential chromatin accessibility. The position weight matrices highlight the different motif sequences (right). The dotted line represents the significance threshold. (B) Differentially enriched gene groups located in cis-co-accessible regions in adipocytes. The score is calculated using -log10(*p*-value). (C) Epigenetic alteration around co-accessible sites enriched in signatures associated with metabolism, cell cycle, and migration. The normalized signal indicates chromatin accessibility, and links identify co-accessible sites. The gray area denotes rearranged chromatins depending on IL-23 or IMQ treatment. Enriched genes are marked in red. Enriched motifs between treatments are displayed above the chromatin immunoprecipitation (ChIP) loci. The bar plot at the bottom shows quantitative real-time PCR results from ChIP assays targeting co-accessible regions containing enriched-motifs, using matched adipose tissues from IL-23- or IMQ-treated samples (n = 3). Data are presented as the mean ± SD. *p < 0.05 using the unpaired *t*-test.

Because epigenomic alterations influence gene expression 33, we further explored DARs in adipocytes. Motif analysis revealed the enrichment of *Klf*/*Sp/Egr1* motifs associated with both positive and negative adipocyte development 34 in the accessible chromatin regions of imiquimod-treated adipocytes (**Figure 6A**). In contrast, IL-23-treated adipocytes harbored motifs related to pluripotency (*Pou5f1, Pou4f2, Pou3f1,* and *Sox2*) 35, adipogenesis (*Nfatc2* and *Nfat5*) 36, 37, and obesity-associated metabolism (*Irf1 and Nr3c1*) 38 (**Figure 6A**). We also compared the co-accessible regions between treatments to identify the cis-regulatory regions and neighboring genes. Adipocytes from imiquimod-treated obese mice showed increased co-accessibility near gene clusters involved in fatty acid oxidation, particularly *Ascl5*, *Tysnd1*, and *Mlycd* (**Figure 6B**-**C**) 39-41. Conversely, cis-regulatory regions near genes associated with the cell cycle and migration were more prevalent in adipocytes from IL-23-treated obese mice (**Figure 6B**-**C**). We further evaluated co-accessible regions, which represent topologically associated genes and regulatory elements with shared transcription factors, through chromatin immunoprecipitation (ChIP) assays targeting transcription factors identified through a motif analysis. Transcription factors, such as *Klf4*, *Sp1*, and *Egr1,* were recruited to chromatin loci near gene clusters involved in fatty acid oxidation in adipocytes from imiquimod-treated obese mice but showed reduced binding in the IL-23-treated group. In contrast, *Pou3f1*, *Pou4f2*, and *Nr3c1* exhibited greater recruitment to chromatin in adipocytes from IL-23-treated obese mice than from those treated with imiquimod (**Figure 6C**). Collectively, these findings suggest that imiquimod-induced systemic inflammation promotes chromatin remodeling associated with enhanced metabolic activity in adipocytes.

### Lipid-associated macrophage subset is decreased in adipose tissue of obese mice with imiquimod-induced psoriatic dermatitis

**Figure 7:**
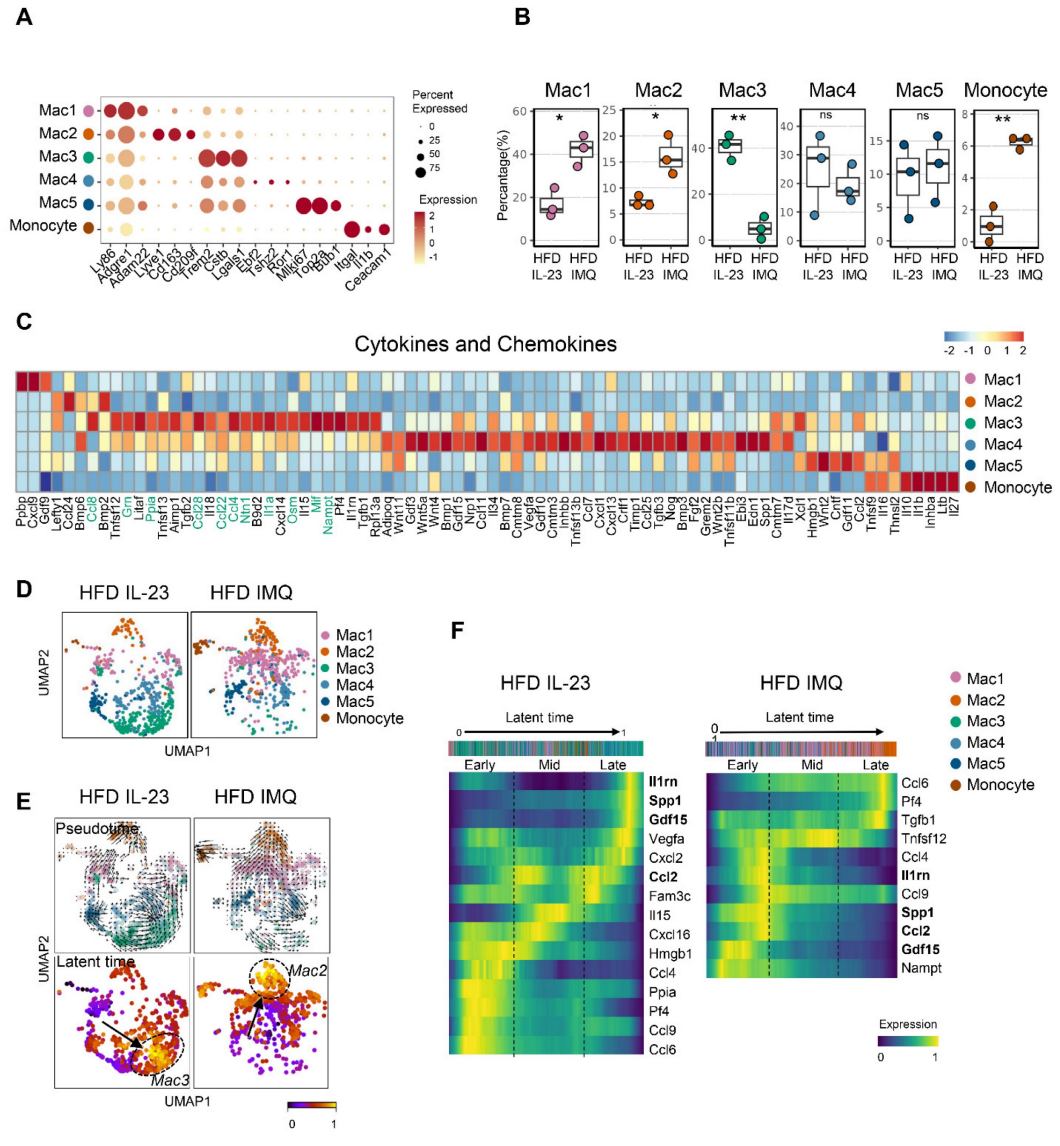
** Lipid-associated macrophages are decreased, while monocytes are increased, in adipose tissue of obese mice with imiquimod-induced psoriatic dermatitis.** (A) Dotplot identification of adipose tissue macrophage subsets by differentially expressed genes across clusters. (B) Differential distributions of adipose tissue macrophage subsets. (C) Heatmap of the expressions of cytokines and chemokines in adipose tissue macrophage subsets. (D) Uniform Manifold Approximation and Projection (UMAP) of the composition of the adipose tissue macrophage subset. (E) Pseudotime was calculated using Velocyto algorithms and is denoted by arrows with directions (upper). Latent time was calculated using scVelo algorithms; differentiation of direction is denoted by score-converted colors (bottom). (F) Gene expression kinetics along the latent time of treatment-dependent trajectories in macrophages. Heatmap indicating gene expression scores of cells according to early, mid, or late latent time. Genes shown in bold indicate overlap between the two treatments, highlighting differences in treatment-specific expression. Data are presented as the mean ± SD. ns: *p* > 0.5, *p < 0.05, and **p < 0.01 using the unpaired *t*-test.

Given the multiple macrophage compartments in the adipose tissue 42, we subdivided them into five subgroups (**Figure 7A**). Notably, macrophage-1 (Mac1) and Mac2 levels increased in the adipose tissue of imiquimod-treated obese mice (**Figure 7B**). Mac1 was characterized by *Ly86* and *Adam22* expression, which are associated with non-classical monocytes involved in vascular integrity 43, and Mac2 expressed markers of perivascular macrophages, such as *Lyve1*, *Cd163*, and *Cd209f* (**Figure 7A**) 44. In contrast, the Mac3 subset, marked by *Trem2*, a sensor of tissue-level lipid homeostasis 42, was decreased in the imiquimod-treated group (**Figure 7B**-**C**). Upregulation of genes linked to adipocyte maturation, such as *Grn*, *Ppia*, *Ntn1*, and *Nampt* 45-48, was observed only in Mac3 cells (**Figure 7C**). Furthermore, the adipose tissue of imiquimod-treated obese mice exhibited increased expression levels of Mac1 and Mac2 marker genes, whereas Mac3 marker genes were upregulated in IL-23-injected mice (**Figure S6**). Interestingly, the macrophage composition shifted from Mac3 to Mac1 and Mac2 in imiquimod-treated mice. In contrast, the reverse was observed in the IL-23-injected group (**Figure 7D**). Furthermore, pseudotime analysis revealed differentiation trajectories toward Mac3 in IL-23-injected obese mice and toward Mac2 in the imiquimod-treated group, with distinct gene expression profiles (**Figure 7E**-**F**).

### Intercellular interactions of adipose tissue macrophages are impaired in obese mice with imiquimod-induced psoriatic dermatitis

**Figure 8:**
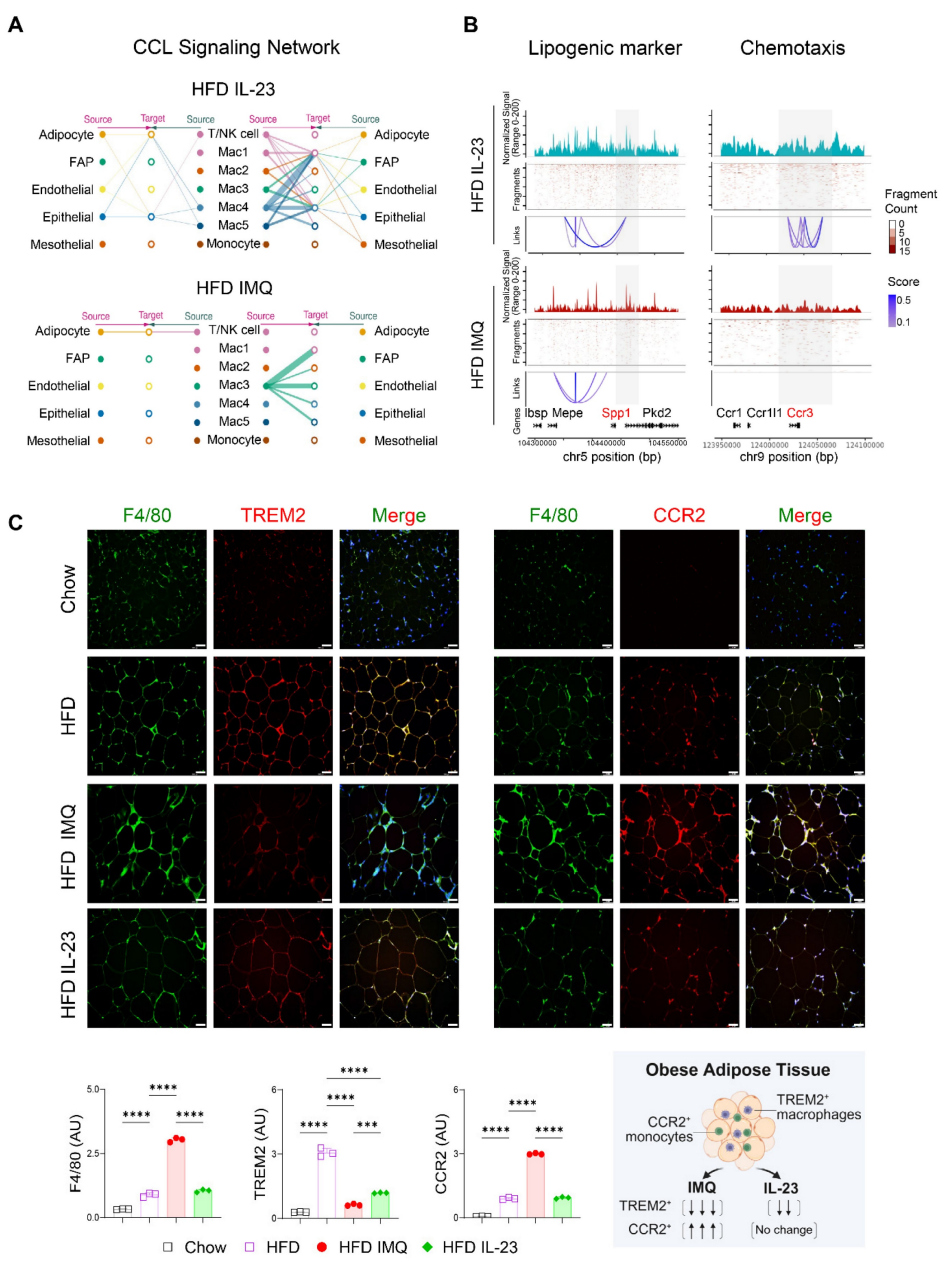
** Chemokine signaling and lipid-associated macrophage dynamics are disrupted in adipose tissue of obese mice with imiquimod (IMQ)-induced psoriatic dermatitis.** (A) CC chemokine ligand (CCL) signaling networks among cell types in adipose tissue. Line connection displays source-target interactions anticipated by ligand and receptor expression profiles. (B) Epigenetic alteration around genes in adipose tissue macrophages. Normalized signal indicates chromatin accessibility, and links indicate co-accessible regions. The gray area represents rearranged chromatins depending on IL-23 or IMQ treatment. Enriched genes are marked as red. (C) Immunofluorescence staining images (top), fluorescence signal quantification (bottom left), and schematic diagram of adipose tissue macrophage subsets (bottom right) in perigonadal adipose tissue. The fluorescence intensity of each section was analyzed. Scale bars = 124.5 μm. Data are presented as the mean ± SD. ****p < 0.0001 using one-way ANOVA.

The notable decrease in chemokine-encoding genes, such as *Ccl8*, *Ccl28*, *Ccl22*, and *Ccl4*, in Mac1 and Mac2 subsets, compared with Mac3 (**Figure 7C**), suggests a reduced intercellular interaction between macrophages and other cell subsets in the adipose tissue of obese mice treated with imiquimod. Network centrality analysis of the IL-23-injected group showed that macrophage clusters were the predominant source of CCL chemokine signaling, functioning in both autocrine and paracrine manners (**Figure 8A**). However, in imiquimod-treated mice, these interactions were absent, and only the Mac3 subset maintained CCL signaling interactions (**Figure 8A**). The chromatin status around genes related to lipid-associated macrophages (*Spp1*) and chemokine signaling (*Ccr3*) further supported the reduced transcription factor accessibility in adipose tissue macrophages of imiquimod-treated obese mice (**Figure 8B**). Immunofluorescence staining also confirmed sustained F4/80^+^TREM2^+^ cell accumulation following IL-23 injection, whereas imiquimod accelerated the HFD-induced increase in F4/80^+^CCR2^+^ monocytes in the adipose tissue of obese mice (**Figure 8C**).

## Discussion

We demonstrated that disruption of the homeostasis of obese adipose tissue, triggered by systemic inflammatory responses in psoriasis, plays a crucial role in exacerbating psoriasis in obesity. Imiquimod-induced psoriatic dermatitis, combined with systemic inflammation, reorganizes chromatin signatures in adipocytes to promote catabolic metabolism and causes a shift in the macrophage subset in the adipose tissue. This shift moved from a subgroup adapted to obesity-associated environmental changes to the accumulation of recruited monocytes lacking intercellular communication capacities. Obesity-associated chronic inflammation upregulates cytokine receptors in adipose tissue 49, making it more susceptible to imiquimod-induced systemic increases in inflammatory cytokines. Additionally, the enlarged adipocytes in obese mice reach a threshold for anti-inflammatory cell removal 50, which may facilitate adipocyte damage in obese mice with psoriatic inflammation. Unlike small lipid droplets, which are easily cleared by adipose tissue macrophages, large lipid droplets resist phagocytosis, leading to CLS formation, in which macrophages surround dead or dying adipocytes and produce inflammatory cytokines 26. The increased presence of CLS in the perigonadal adipose tissue of imiquimod-treated obese mice suggests that recruited monocytes contribute to the increased formation of CLS. This was further supported by an increase in the monocyte subset, as revealed by single-nucleus RNA-seq of perigonadal adipose tissue from imiquimod-treated obese mice.

Our data indicate that obesity indirectly exacerbates psoriasis by increasing the susceptibility to adipose tissue damage in response to systemic inflammatory triggers. Previous studies using an imiquimod-induced murine psoriasis model highlighted the direct role of obesity or obesity-induced dyslipidemia in exacerbating psoriasis 5, 51. However, mice exhibit lower levels of atherogenic lipoproteins than those in humans and have a lower risk of dyslipidemia than those of other species, such as rabbits or hamsters 52, as reflected by the decreased triglyceride levels observed in obese mice in the present study. Therefore, caution is warranted when interpreting the causal relationship between serum lipid profiles and psoriasis severity using murine models. Furthermore, we observed that psoriatic dermatitis induced by intradermal IL-23 injection, which had no apparent systemic influence, did not aggravate dermatitis in obese mice, despite the presence of obesity-associated metabolic and pro-inflammatory changes. While obesity increases the risk of metabolic disease, many obese individuals remain resistant to metabolic syndrome and maintain nutrient homeostasis through efficient energy stores 10, 53. The improved metabolic profile of genetically engineered mice, which preferentially store triglycerides in white adipose tissue, illustrates the importance of healthy adipocytes 54. However, the inflammatory microenvironment of adipose tissue, accompanied by hypoxic enlargement of adipose tissue 55 and chronic inflammatory diseases, accelerates adipocyte death in obesity 56. Consistently, the systemic elevation of multiple pro-inflammatory cytokines during the progression of psoriasis may contribute to adipose tissue damage, thereby promoting comorbidities associated with obesity. Although the systemic administration of IL-23 was not evaluated in this study, subcutaneous IL-23 injection induces psoriatic inflammation comparable to that triggered by imiquimod 57. Moreover, the systemic overexpression of IL-23 in mice fed a Western diet, characterized by high sugar and moderate fat, induces more pronounced psoriatic inflammation than that for a HFD and promotes both skin and joint inflammation 58, 59. Therefore, differences in dietary composition and the route of IL-23 administration should be carefully considered when comparing inflammatory responses in lean and obese mice subjected to IL-23 injection.

Epigenetic regulation has been implicated in the occurrence and progression of metabolic diseases 60, and variations in DNA methylation and histone modifications have been associated with obesity-driven metabolic disturbance 61. Our single-nucleus multiome approach identified epigenetically different adipocytes with matched gene expression profiles at single-cell resolution and revealed metabolic impairments upon imiquimod treatment. Although live and intact single-nucleus preparations are limited to the detection of damaged adipocytes, imiquimod-driven systematic inflammatory responses reorganize accessible chromatin regions and enhance the expression of selected gene sets required for fat metabolism. Although our study provides insights into how imiquimod-induced systemic inflammation remodels adipose tissue in obese mice, the lack of an untreated obese control group in our single-nucleus sequencing analyses limits our ability to distinguish psoriasis-specific effects from baseline obesity-driven changes. Obesity alone is known to induce transcriptional and epigenetic reprogramming in adipose tissues 16, 62, and our findings suggest that systemic inflammation is associated with additional regulatory processes that disrupt adipocyte homeostasis and alter the macrophage composition. Future studies incorporating untreated obese mice will be critical to define these effects. Moreover, epigenetic profiling of DNA methylation and histone modifications in adipocytes and macrophages may help identify inflammation-constrained cell states and inform the development of strategies to restore adipose tissue function under chronic inflammatory conditions.

Obese individuals exhibit increased adipocyte-macrophage communication and receptor-ligand interaction-related gene expression within adipocytes and other cell subsets in adipose tissue 23. Notably, although the adipose tissues of obese individuals exhibit upregulated inflammatory cytokines coupled with enriched immune cells 63, certain levels of controlled inflammation are required for adipose tissue remodeling and maintenance of metabolic homeostasis 14. Accordingly, adipose tissue macrophages play a critical role in maintaining lipid homeostasis, even in inflammatory phenotypes 64. Chemokines and inflammatory mediators released by adipose tissue macrophages recruit adipocyte progenitors to promote their differentiation into mature adipocytes 63. The Mac3 subset, with upregulated expression of genes critical for lipid homeostasis and inflammatory responses was markedly decreased in the adipose tissue of imiquimod-treated obese mice. Notably, the Mac3 subset displayed increased *Trem2* expression, a gene encoding a lipid receptor that supports lipid homeostasis under obese conditions 42. In contrast, the Mac1 and Mac2 subsets, which were more prevalent in the adipose tissue of imiquimod-treated obese mice, exhibited downregulated genes that are characteristic of the lipid-associated macrophage signature, accompanied by decreased chromatin accessibility around the genes responsible for lipid handling and inflammatory migration, suggesting a failure to maintain lipid homeostasis and regulate metabolic inflammation, potentially exacerbating obesity-associated inflammatory responses in psoriasis. Given that metabolically activated macrophages exhibit limited responsiveness to classical pro-inflammatory stimuli due to their distinct lipid-handling and metabolic programming, rather than the engagement of conventional pathogen-sensing pathways 65, the distinct shifts in macrophage populations between imiquimod and IL-23 treatment may underlie the decreased *S100a9* and *Il1b* expression observed in the adipose tissue of IL-23-treated obese mice.

Although single-nucleus RNA-seq revealed predominant expression of *Trem2* in the Mac3 subset of obese mice injected with IL-23, the protein level of TREM2 in the perigonadal adipose tissue of these mice was significantly decreased compared with that in untreated obese mice. This discrepancy might originate from potential unidentified immune microenvironmental changes in the adipose tissue of obese mice injected with IL-23. Additionally, we did not observe changes in adipose tissue in obese patients with psoriasis. To elucidate the bidirectional pathogenic communication between the skin and adipose tissue, further investigations are necessary, particularly to confirm the differences between obese adipose tissue unaffected by psoriasis and that with varying degrees of psoriatic inflammation.

## Conclusions

Obesity sensitizes adipose tissue to homeostatic disruption, in which dysregulated adipocyte function and increased inflammatory responses exacerbate psoriasis under systemic inflammatory conditions. Imiquimod-induced inflammation drives epigenetic remodeling in adipocytes and shifts macrophage populations toward pro-inflammatory states, impairing lipid handling and intercellular communication. These findings identify adipose tissue as a critical immunometabolic interface and suggest that preserving its integrity and attenuating systemic inflammation may help regulate the exacerbation of obesity-associated psoriasis.

## Supplementary Material

Supplementary figures and tables.

## Abbreviations

ATAC: assay for transposase-accessible chromatin
BSA: bovine serum albumin
c-caspase-3: cleaved-caspase-3
CCL: C-C motif ligand
cDNA: complementary DNA
ChIP: chromatin immunoprecipitation
CLS: crown-like structure
CXCL: C-X-C motif ligand
DAR: differentially accessible region
DEG: differentially expressed gene
FAP: fibroblast and adipocyte progenitors
GSEA: gene set enrichment analysis
HFD: high-fat diet
HO-1: heme oxygenase-1
HTO: hash-tag barcoding antibody-oligo
PBS: phosphate-buffered saline
pRIP3: phosphorylated receptor-interacting protein kinase 3
RNA-seq: RNA-sequencing
TNFα: tumor necrosis factor-alpha
UMAP: Uniform Manifold Approximation and Projection
