# Supplementary material for: Systemic inflammation-induced adipose tissue remodeling drives psoriasis exacerbation in obesity through epigenetic and immunometabolic dysregulation
Source: Theranostics. 2025 Jul 28;15(16):8639–57. doi: 10.7150/thno.116796 (PMC12374732; doi:10.7150/thno.116796)
Supplement: Supplementary file 1 — Supplementary figures and tables. [file thnov15p8639s1.pdf]

Supplemental materials

1. Supplemental Methods

Liquid chromatography–tandem mass spectrometry (LC–MS/MS) analysis

LC–MS/MS was performed using an Agilent HPLC 1200 system (Agilent Technologies, Santa Clara, CA, USA) coupled to an AB SCIEX Triple Quad 5500 mass spectrometer equipped with a turbo ion spray interface in positive ionization mode (Applied Biosystems-SCIEX, Concord, ON, Canada). Chromatography was performed in the isocratic mode using a mobile phase consisting of 0.1% formic acid in water and acetonitrile (30:70, v/v) at a flow rate of 0.2 mL/min and the Synergi™ 4 µm polar-RP 80A column (75 × 2.0 mm, 2.6 µm; Phenomenex, Torrance, CA, USA) at 30 °C. The autosampler was maintained at 4 °C, and the injection volume was 10 µL for mouse plasma and tissue samples. Selected reaction monitoring (SRM) transitions were *m/z* 241.0 → 168.0 for imiquimod and 180.2 → 162.2 for the phenacetin internal standard. Mass data were processed using Analyst software version 1.5.2 (Applied Biosystems-SCIEX). The limit of quantitation was 1 ng/mL for imiquimod.

Enzyme-linked immunosorbent assay (ELISA)

A Quantikine Mouse IL-23 ELISA Kit (R&D Systems, Minneapolis, MN, USA) was used to assess IL-23 following the manufacturer's instructions. The absorbance was read at 450 nm using a VARIOSKAN LUX instrument (Thermo Fisher Scientific, Waltham, MA, USA).

Assessment of cutaneous blood perfusion

Skin blood perfusion in mice treated topically with imiquimod or injected intradermally with IL-23 was monitored using a laser Doppler perfusion imaging system (Moor Instruments, Devon, UK). Perfusion levels are presented in arbitrary units. For analyses, representative images from one mouse per group were used, with regions of interest defined based on the dorsal skin area.

2. Supplemental Figures

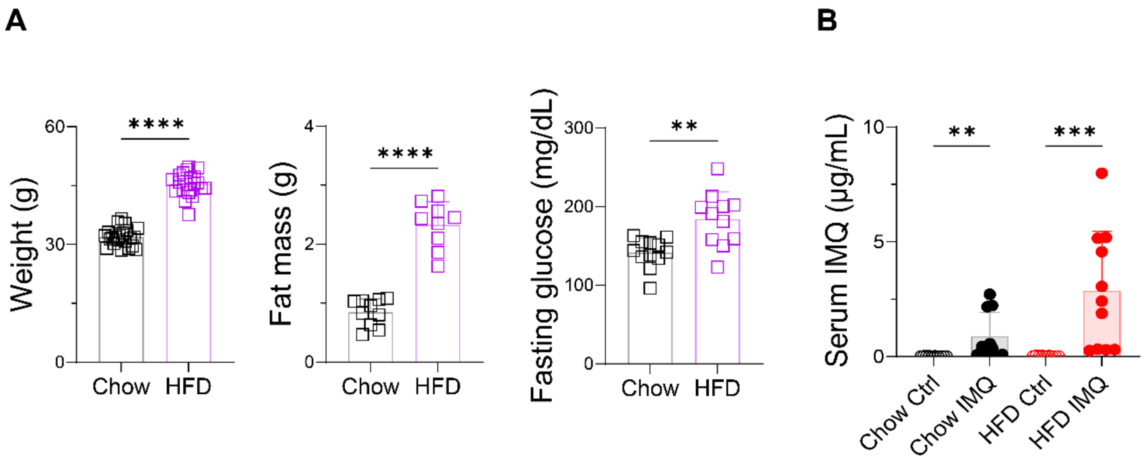

Figure S1. Obesity-associated parameters in mice fed a high-fat diet (HFD). (A) Body weight, perigonadal adipose tissue mass, and fasting glucose levels in mice. Fasting glucose was measured using a glucometer (Allmedicus, Kyunggi, Korea) following starvation for 18 h. (B) Serum levels of imiquimod (IMQ). Data are presented as the mean ± SD. $^{**}p < 0.01$, $^{***}p < 0.001$, and $^{****}p < 0.0001$ using the unpaired *t*-test (A), Mann–Whitney test (fat mass in A), or Kruskal–Wallis test (B).

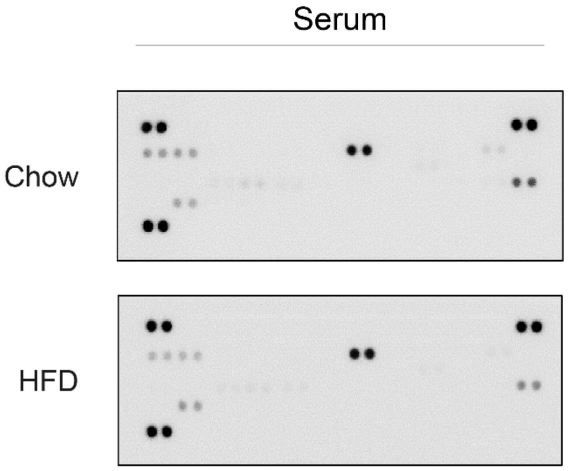

Figure S2. Serum cytokine profiles in mice. Forty different cytokines were semi-quantified from mouse serum using a cytokine array.

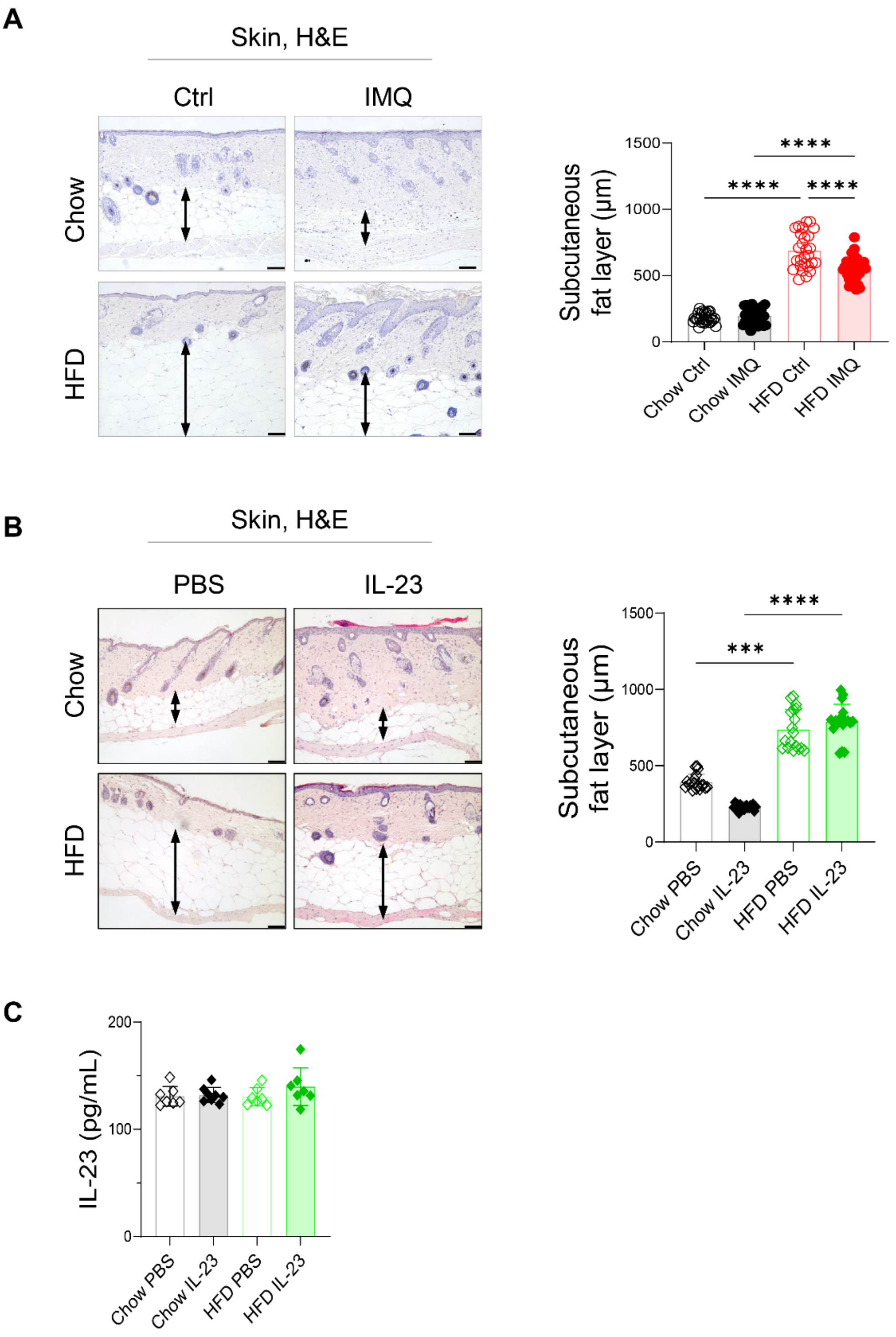

Figure S3. Subcutaneous fat thickness and serum IL-23 levels in obese mice with psoriatic dermatitis induced by imiquimod (IMQ) or IL-23. (A and B) Hematoxylin and eosin-stained images (left) and the quantification of subcutaneous fat thickness (right) in mice treated with IMQ (A) and IL-23 (B). (C) Serum levels of IL-23. Data are presented as the mean ± SD. ***$p < 0.001$ and ****$p < 0.0001$ using one-way ANOVA (A) or Kruskal–Wallis test (B).

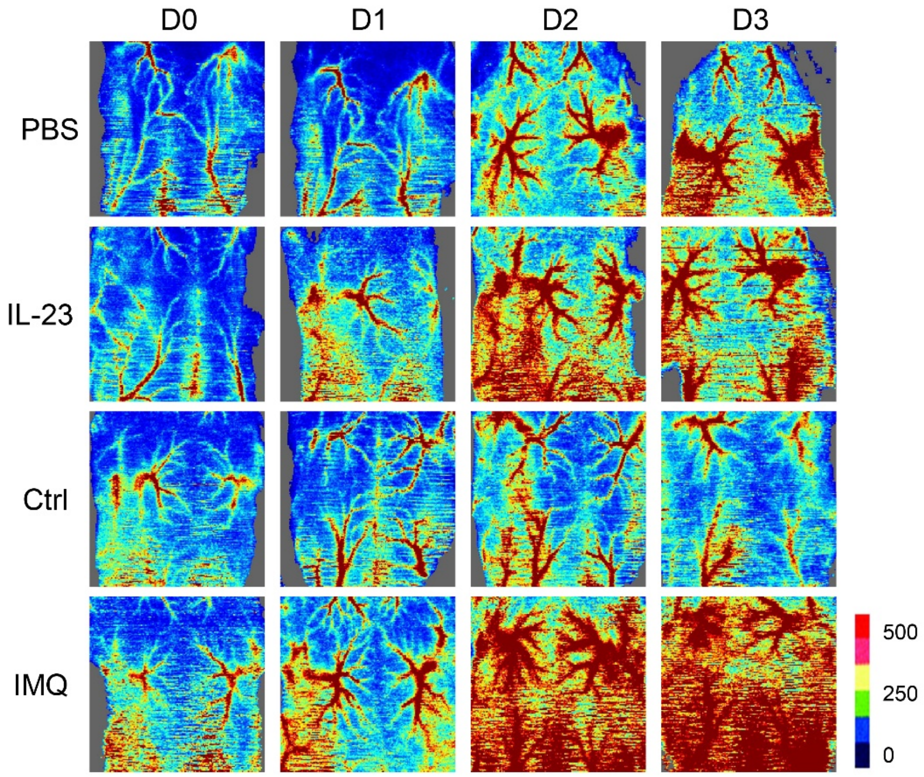

Figure S4. Cutaneous blood perfusion. Representative images showing blood perfusion in the dorsal skin of mice treated with imiquimod (IMQ) or IL-23. Images for the vehicle (Ctrl) and IMQ groups were previously published by Kim *et al*. [1] and are reused here under an open-access license (CC BY).

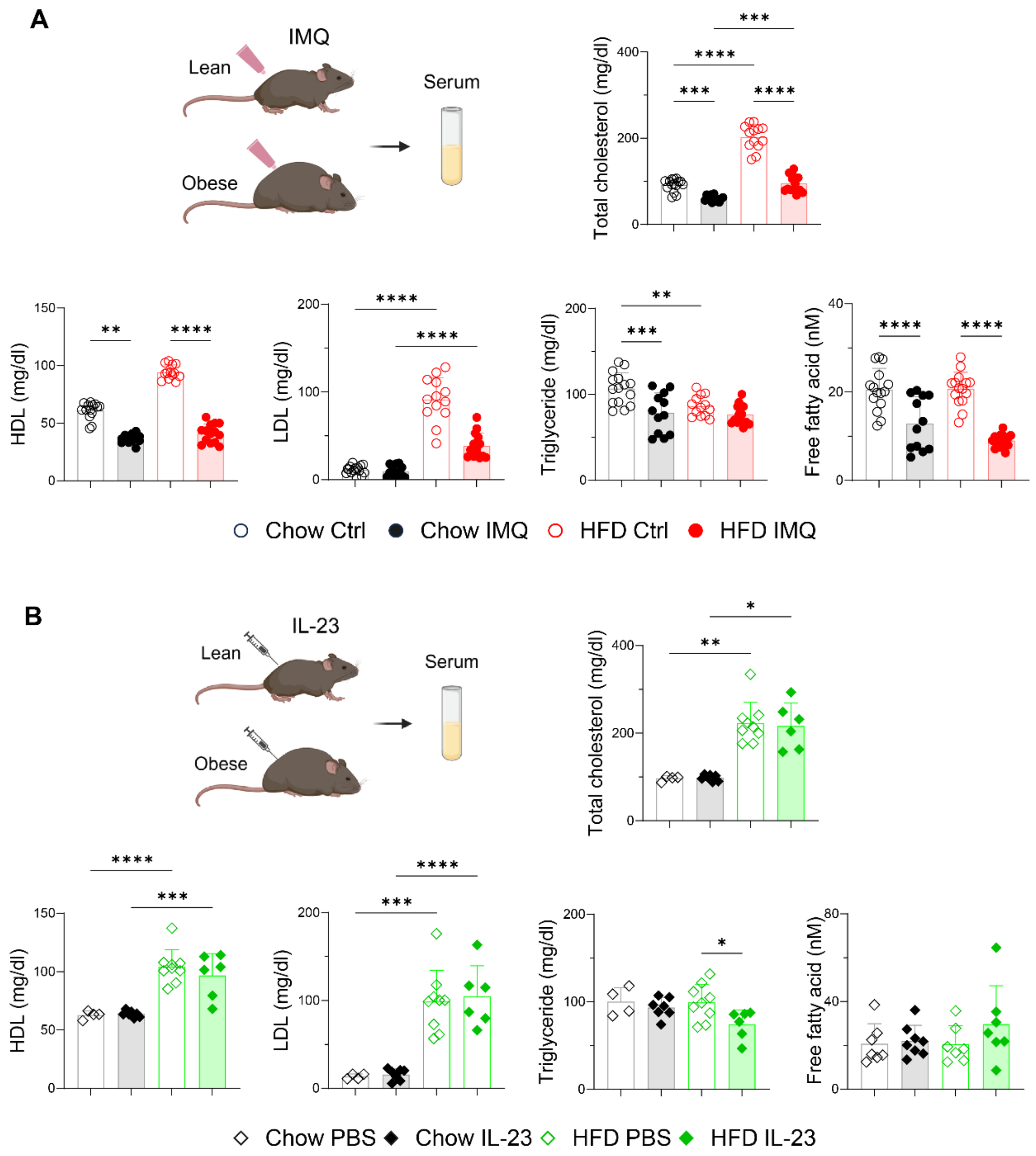

Figure S5. Serum lipid profiles in mice with psoriatic inflammation. (A and B) Serum levels of total cholesterol, high-density lipoprotein (HDL), low-density lipoprotein (LDL), triglyceride, and free fatty acid in mice treated with imiquimod (IMQ; A) and IL-23 (B). Total cholesterol, HDL, LDL, and triglyceride were measured using an AU480 Chemistry Analyzer (Beckman Coulter, Brea, CA, USA). Free fatty acid was measured using the Free Fatty Acid Quantitation Kit (Sigma-Aldrich, St. Louis, MO, USA) following the manufacturer's instructions. Data are presented as the mean ± SD. $^{**}p < 0.01$, $^{***}p < 0.001$, and $^{****}p < 0.0001$ using one-way ANOVA or Kruskal–Wallis test (HDL in A).

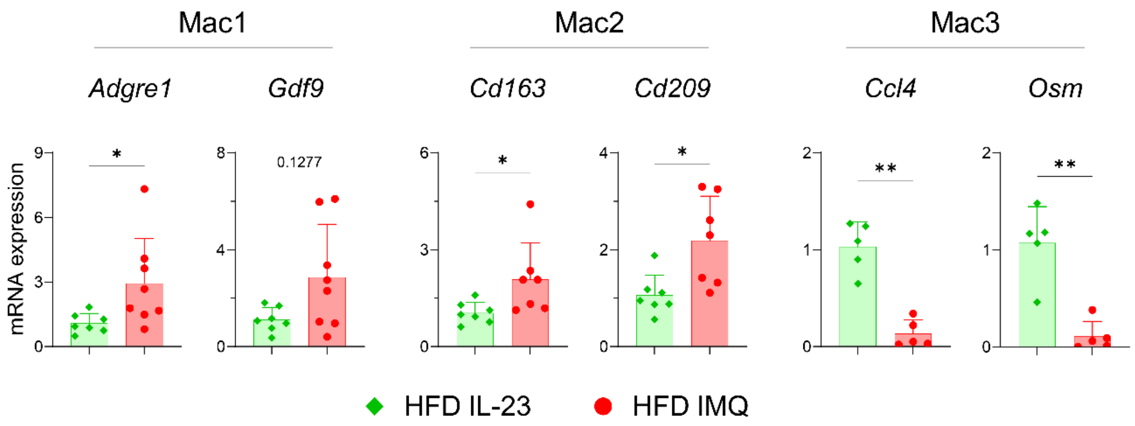

Figure S6. Quantitative PCR analysis of differentially expressed genes representing macrophage subsets in adipose tissue. Data are presented as the mean ± SD. *$p < 0.05$ and **$p < 0.01$ using the unpaired *t*-test or Mann–Whitney test (*Adgre 1* and *Gdf9*).

3. Supplemental Tables

Table S1. Primer sequences for real-time quantitative PCR

Target gene	Primer sequence
S100a8	Forward: 5′-TTC CTT GCG ATG GTG ATA-3′<br>Reverse: 5′-ATG ATG ACT TTA TTC TGT AGA CA-3′
S100a9	Forward: 5′-TGA GGA GTG TAT GAT GCT GAT G-3′<br>Reverse: 5′-CAT TCC CTT TAG ACT TGG TTG G-3′
Il1b	Forward: 5′-GCA ACT GTT CCT GAA CTC AAC T-3′<br>Reverse: 5′-ATC TTT TGG GGT CCG TCA AC-3′
Tnf	Forward: 5′-CCT GTA GCC CAC GTC GTA G-3′<br>Reverse: 5′-GGG AGT AGA CAA GGT ACA ACC C-3′
Cxcl1	Forward: 5′-AGT CAT AGC CAC ACT CAA GAA T-3′<br>Reverse: 5′-TCA GAA GCC AGC GTT CAC-3′
Il22	Forward: 5′-ACC AGA ACA TCC AGA AGA AT-3′<br>Reverse: 5′-CTC AGA CGC AAG CAT TTC-3′
Il23	Forward: 5′-CTA AGA GAA GAA GAG GAT GAA GAG-3′<br>Reverse: 5′-CTG GCT GTT GTC CTT GAG-3′
Il17a	Forward: 5′-GAC TTC CTC CAG AAT GTG AA-3′<br>Reverse: 5′-TGG AAC GGT TGA GGT AGT-3′
Adgre1	Forward: 5′-CAA CTG CTC TAA CTC TGT GGG-3′<br>Reverse: 5′-GTG GCA GGT TGC ATG TTC AG-3′
Gdf9	Forward: 5′-TGG AAC ACT TGC TCA AAT CGG-3′<br>Reverse: 5′-GAC ATG GCC TCC TTT ACC ACA-3′
Cd163	Forward: 5′-GGT GGA CAC AGA ATG GTT CTT C-3′<br>Reverse: 5′-CCA GGA GCG TTA GTG ACA GC-3′
Cd209	Forward: 5′-GAG AAG CAG AGG ATG GCA GG-3′<br>Reverse: 5′-TCC CTG CTC TCC AGA TGT GA-3′
Ccl4	Forward: 5′-TTC CTG CTG TTT CTC TTA CAC CT-3′<br>Reverse: 5′-CTG TCT GCC TCT TTT GGT CAG-3′
Osm	Forward: 5′-GAC AG CAT AGT CTG GTG ATA CA-3′<br>Reverse: 5′-CCT TGA AGT CCT GAG TGG TAG-3′
Gapdh	Forward: 5′-CTG GTA TGA CAA TGA ATA CGG-3′<br>Reverse: 5′-GCA GCG AAC TTT ATT GAT GG-3′

Table S2. Oligonucleotides sequences for hashing of nuclei and construction of cell hashing library

Oligonucleotides	Sequence	Source
TotalSeq™-A0451 anti-Nuclear Pore Complex Proteins Hashtag 1 Antibody	TTCCTGCCATTACTA	BioLegend
TotalSeq™-A0452 anti-Nuclear Pore Complex Proteins Hashtag 2 Antibody	CCGTACCTCATTGTT	BioLegend
TotalSeq™-A0453 anti-Nuclear Pore Complex Proteins Hashtag 3 Antibody	GGTAGATGTCCTCAG	BioLegend
TotalSeq™-A0454 anti-Nuclear Pore Complex Proteins Hashtag 4 Antibody	TGGTGTCATTCTTGA	BioLegend
TotalSeq™-A0455 anti-Nuclear Pore Complex Proteins Hashtag 5 Antibody	ATGATGAACAGCCAG	BioLegend
TotalSeq™-A0456 anti-Nuclear Pore Complex Proteins Hashtag 6 Antibody	CTCGAACGCTTATCG	BioLegend
TotalSeq™-A0457 anti-Nuclear Pore Complex Proteins Hashtag 7 Antibody	CTTATCACCGCTCAA	BioLegend
TotalSeq™-A0458 anti-Nuclear Pore Complex Proteins Hashtag 8 Antibody	TGACGCCGTTGTTGT	BioLegend
HTO Cell Hashing cDNA PCR additive primer	GTGACTGGAGTTCAGACGTGTGCTC	BioLegend

Table S3. Antibodies for chromatin immunoprecipitation

Transcription Factor	Product Number	Product Name
POU4F2	ab317492	Anti-BRN3A + BRN3B + BRN3C antibody [EPR26313-54]
POU3F1	ab259952	Anti-Oct6 antibody [EPR24057-94]
NR3C1	ab183127	Anti-Glucocorticoid Receptor antibody [EPR19621]
KLF5	ab137676	Anti-KLF5 antibody
SP1	ab227383	Anti-SP1 antibody
EGR1	ab300449	Anti-Egr1 antibody [EPR23981-46]

Table S4. Primer sequences for quantitative PCR

Target region	Primer sequence
Chr19:55100177-55100285	Forward: 5′-ATC CAT CGG TGT CAG ACT GC-3′
(*Acsl5-associated locus*)	Reverse: 5′-TTG TGC CTC AGC TCC CAG TA-3′
Chr10:61648826-61648944	Forward: 5′-TTC ATT CCA GAA CGA GCG GG-3′
(*Tyrnd1-associated locus*)	Reverse: 5′-TTC GAA TGT GTG GAG GGA GC-3′
Chr8:119394560-119394685	Forward: 5′-GAA CCC TAG CAC AGG AGG C -3′
(*Mylcd-associated locus*)	Reverse: 5′-CCT GTT CTG AGA GTC ACC GC -3′
Chr14:50892963-50893073	Forward: 5′-AGG TAA TCG TGG GCT TTC CC-3′
(*Apex1-associated locus*)	Reverse: 5′-GTG TCA GAA AGC CTT GGC AC-3′
Chr5:137045770-137045896	Forward: 5′-CGC CAC CAT TCA GCT CTA GT-3′
(*Serpine-associated locus-1*)	Reverse: 5′-CGC AAA GGA TCC ACT TCC TG-3′
Chr5:137071904-137072013	Forward: 5′-TGG GGA AGG ACC CAA AGA CT-3′
(*Serpine-associated locus-2*)	Reverse: 5′-TTA GTC CAC CCT AGC CCA GC-3′

Reference

1. Kim HJ, Jang J, Na K, Lee E-H, Gu H-J, Lim YH, et al. TLR7-dependent eosinophil degranulation links psoriatic skin inflammation to small intestinal inflammatory changes in mice. Exp Mol Med. 2024; 56: 1164-77.
